# Interactive effect of *STAT6* and *IL13* gene polymorphisms on eczema status: results from a longitudinal and a cross-sectional study

**DOI:** 10.1186/1471-2350-14-67

**Published:** 2013-07-02

**Authors:** Ali H Ziyab, Gwyneth A Davies, Susan Ewart, Julian M Hopkin, Eric M Schauberger, Marsha Wills-Karp, John W Holloway, Syed Hasan Arshad, Hongmei Zhang, Wilfried Karmaus

**Affiliations:** 1 Department of Epidemiology and Biostatistics, Norman J. Arnold School of Public Health, University of South Carolina, Columbia, South Carolina, USA; 2 Department of Community Medicine and Behavioral Sciences, Faculty of Medicine, Kuwait University, Kuwait, Kuwait; 3 College of Medicine, Swansea University, Swansea, Wales, UK; 4 College of Veterinary Medicine, Michigan State University, East Lansing, Michigan, USA; 5 Department of Pediatrics, Medical College of Wisconsin, Milwaukee, Wisconsin, USA; 6 Department of Environmental Health Sciences, Bloomberg School of Public Health, Johns Hopkins University, Baltimore, Maryland, USA; 7 Academic Units of Clinical and Experimental Medicine, Faculty of Medicine, University of Southampton, Southampton, UK; 8 Human Development and Health, Faculty of Medicine, University of Southampton, Southampton, UK; 9 David Hide Asthma and Allergy Research Centre, Isle of Wight, UK; 10 Division of Epidemiology, Biostatistics, and Environmental Health School of Public Health, University of Memphis, Memphis, TN, USA

**Keywords:** Eczema, Gene-gene Interaction, Epistasis, STAT6, IL13, Genetic Association Study

## Abstract

**Background:**

Eczema is a prevalent skin disease that is mainly characterized by systemic deviation of immune response and defective epidermal barrier. Th2 cytokines, such as IL-13 and transcription factor STAT6 are key elements in the inflammatory response that characterize allergic disorders, including eczema. Previous genetic association studies showed inconsistent results for the association of single nucleotide polymorphisms (SNPs) with eczema. Our aim was to investigate whether SNPs in *IL13* and *STAT6* genes, which share a biological pathway, have an interactive effect on eczema risk.

**Methods:**

Data from two independent population-based studies were analyzed, namely the Isle of Wight birth cohort study (IOW; n = 1,456) and for the purpose of replication the Swansea PAPA (Poblogaeth Asthma Prifysgol Abertawe; n = 1,445) cross-sectional study. Log-binomial regressions were applied to (i) account for the interaction between *IL13* (rs20541) and *STAT6* (rs1059513) polymorphisms and (ii) estimate the combined effect, in terms of risk ratios (RRs), of both risk factors on the risk of eczema.

**Results:**

Under a dominant genetic model, the interaction term [*IL13* (rs20541) × *STAT6* (rs1059513)] was statistically significant in both studies (IOW: adjusted *P*_interaction_ = 0.046; PAPA: *P*_interaction_ = 0.037). The assessment of the combined effect associated with having risk genotypes in both SNPs yielded a 1.52-fold increased risk of eczema in the IOW study (95% confidence interval (CI): 1.05 – 2.20; *P* = 0.028) and a 2.01-fold higher risk of eczema (95% CI: 1.29 – 3.12; *P* = 0.002) in the PAPA study population.

**Conclusions:**

Our study adds to the current knowledge of genetic susceptibility by demonstrating for the first time an interactive effect between SNPs in *IL13* (rs20541) and *STAT6* (rs1059513) on the occurrence of eczema in two independent samples. Findings of this report further support the emerging evidence that points toward the existence of genetic effects that occur via complex networks involving gene-gene interactions (epistasis).

## Background

The elevated prevalence of eczema among children (15% to 30%) and adults (2% to 10%) makes this skin disorder a public-health concern by placing heavy economic burden on families and societies [1-3]. Eczema is an inflammatory skin disorder that is characterized by disrupted epidermal barrier function, immunoglobulin E (IgE)-mediated sensitization to food and environmental allergens (i.e., allergic sensitization), and a relapsing–remitting combination of skin dryness and itching [2,4]. The identification of loss-of-function variants in the filaggrin gene (*FLG*) caused a shift in the research paradigm from mainly focusing on immune related genes to genetic factors that regulate the formation of the epidermal barrier [5]. Controversially, however, existing and evolving evidence suggests that type-2 T helper (Th2) cytokines can modulate the expression of proteins that are essential for the formation and integrity of the epidermal barrier [6-8].

Chromosome 5q31-33 harbors genes, including interleukin 4 (*IL4*) and interleukin 13 (*IL13*), that encode Th2 cytokines, which are involved in the regulation of IgE synthesis and manifestation of allergic disorders [1,2,9,10]. Th2 cytokines, IL-4 and IL-13, exert their biological effect through the activation of the transcription factor STAT6 (signal transducer and activator of transcription 6), which is encoded on chromosome 12q13-24 [9]. Briefly, the binding of IL-4/IL-13 to their respective membrane receptor leads to the activation of STAT6 by a phosphorylation process [11,12]. Once activated, STAT6 homodimers rapidly translocate to the nucleus and induce or suppress the transcription of target genes [11-13]. Among the various target genes of STAT6 are the genes located in the epidermal differentiation complex, which refers to a genomic region containing a cluster of genes that are critical for the development of functional skin barrier [6,13,14]. Hence, altered STAT6 activation may contribute to the major hallmarks of eczema: (i) allergic sensitization and (ii) impaired epidermal barrier.

Even though IL-4 and IL-13 share many biological functions and signal through a common receptor subunit (IL-4 receptor α chain), it has been suggested that IL-13 is more important than IL-4 in mediating allergic inflammation [15,16]. Genetic variants within the *IL13* gene have been associated with different allergic phenotypes, with the non-synonymous coding variant rs20541 (R130Q) being the most replicated [9,16]. More importantly, Vladich et al. demonstrated that the *IL13* rs20541 130Q variant is more active than the 130R wild-type in inducing *STAT6* phosphorylation/activation [17,18]. In addition to the role of *IL13* genetic polymorphisms in allergic responses, genetic variants in *STAT6* gene have been linked to multiple allergic phenotypes [19-21]. A recent genome-wide association study (GWAS) showed that a single nucleotide polymorphism (SNP) in *STAT6* (rs1059513; tagging SNP) is associated with serum total IgE levels [22].

Therefore, based on the aforementioned investigations we hypothesize that a complex gene-gene interaction (epistasis) between *IL13* and *STAT6* genes could be important in the pathogenesis of eczema. Our search of the literature indicated a lack of studies investigating the possible interaction between genetic variants in *IL13* and *STAT6* genes in relation to eczema risk. The purpose of this study was to determine whether an interaction between rs20541 in *IL13* and rs1059513 in *STAT6* on eczema risk exists. The investigation was conducted in two independent population-based studies, namely the Isle of Wight birth cohort (IOW) and the Swansea PAPA (Poblogaeth Asthma Prifysgol Abertawe) cross-sectional study.

## Methods

### Isle of Wight study

#### Study population and characteristics

A whole population birth cohort was established on the Isle of Wight, UK, in 1989 to prospectively study the natural history of allergic diseases from birth to 18 years of age. The island is close to the British mainland, semi-rural, and without heavy industry. Both the Isle of Wight and the study population are 99% Caucasian. Ethics approvals were obtained from the Isle of Wight Local Research Ethics Committee (now named the National Research Ethics Service, NRES Committee South Central – Southampton B) at recruitment and for the 1, 2, 4, 10 and 18 years follow-up (06/Q1701/34). Of the 1,536 children born between January 1, 1989, and February 28, 1990, written informed consent was obtained from parents to enrol 1,456 newborns. Children were followed up at the ages of 1 (n = 1,167), 2 (n = 1,174), 4 (n = 1,218), 10 (n = 1,373), and 18 years (n = 1,313). Detailed questionnaires were completed for each child at each follow-up. When a visit was not possible, a telephone questionnaire was completed or a postal questionnaire sent for completion and return.

In all assessments of the Isle of Wight birth cohort, eczema was defined as chronic or chronically relapsing, itchy dermatitis lasting more than 6 weeks with characteristic morphology and distribution [23], following Hanifin and Rajka criteria [24]. Since the 1-year and 2-year follow-up data on eczema were collected in a relatively small time window, we combined them for analytic purposes (reported as 1-or-2 years).

#### Genotyping

DNA was extracted from blood or saliva samples from IOW cohort subjects (n = 1,211). Seven SNPs in the *IL13* gene were selected for genotyping using a tagging strategy implemented in Haploview using HapMap Caucasian data (Hapmap Data PhaseIII/Rel#2, Feb09, on NCBI B36 assembly, dbSNP b126) [25]. One SNP in *STAT6* was selected for replication purposes. DNA samples were interrogated using GoldenGate Genotyping Assays (Illumina, Inc, SanDiego, CA) on the BeadXpress Veracode bead platform (Illumina, Inc, SanDiego, CA) per Illumina’s protocol. Data were analyzed using the genotyping module of the GenomeStudio Software package (Illumina, Inc, SanDiego, CA). DNA from each subject plus 37 replicate samples, genotyped for control purposes, were analyzed for a total of 1,248 samples (trios were not available). The quality threshold for allele determination across samples was set at a GenCall score >0.25 with n = 1,227 samples retained for further analysis. Analysis of each locus included reclustering of genotyping data using our project data to define genotype cluster positions with additional manual reclustering to maximize both cluster separation and the 50th percentile of the distribution of the GenCall scores across all genotypes (50% GC score). GenCall score is a quality metric of the Illumina GenomeStudio software that indicates the reliability of the genotypes called on SNP arrays, with scores ranging from 0.0 to 1.0. The proprietary algorithm for converting raw allele intensities into genotypes considers angle of the clusters, dispersion of clusters, overlap between clusters, and intensity. Genotypes with lower GenCall scores are located furthest from the center of the cluster. For the analyzed *STAT6* and *IL13* SNPs the sample call rates were over 95.5%. GenCall scores and other quality metrics for SNPs rs20541 and rs1059513 are reported in Additional file 1: Table S1.

### Swansea PAPA study

#### Study population and characteristics

PAPA (*Poblogaeth Asthma Prifysgol Abertawe*, Swansea University Asthma Population) is a cross-sectional population-based study. The study group comprised 1,445 unselected Caucasian volunteers (aged 18–30) from students and staff at Swansea University and Singleton Hospital, Swansea, United Kingdom. Eczema was defined by a positive response to ‘Have you ever had eczema?’ and further defined by whether participants currently had symptoms using a validated questionnaire [26].

#### Genotyping

DNA was extracted from blood samples provided by the PAPA population subjects (n = 1445). Eight and seven variants in *IL13* and *STAT6* genes, respectively, that were reported to be common in populations of European ancestry and/or showed potential association with allergic phenotypes were selected for genotyping. In order to ensure sufficient heterozygosity in our population, variant selection was refined by genotyping a small sample of our population for the selected SNPs of interest by automated DNA sequencing (ABI PRISMTM 310 Gene Analyser). Following SNP selection, genotyping of selected SNPs was performed by KBioscience (http://www.kbioscience.co.uk) using a fluorescence-based competitive allele-specific PCR system (KASPar). This proprietary system has been developed from the Ampliflour system. SNP Call rates were over 98% for all variants (Additional file 1: Table S1). A random sample of the population (n = 30) underwent in-house automated DNA sequencing (ABI PRISM 310 Gene Analyzer) with results validated against the overall genotyping results for the population and results were concordant (trios were not available). Randomly selected samples were also analyzed in duplicate by KBioscience to provide further validation. Blank cells were included on each 96-well plate (sterile water) providing negative controls.

### Statistical analysis

Period prevalence of eczema in the IOW study was determined for each follow-up (1-or-2, 4, 10, and 18 years) by dividing the number of participants with eczema by the total number of individuals who participated in the respective follow-up. Similarly, the proportion of participants with eczema in the PAPA study was determined. In both studies, proportion of individuals with eczema was further stratified by *IL13* (rs20541) and *STAT6* (rs1059513) SNP genotypes. In this report we limited our analysis to a single SNP in each gene due to their known relevance to characteristics of the outcome under-investigation (eczema) and their availability in both studies. The *STAT6* tagging SNP rs1059513 is the only common SNP in *STAT6* that had been genotyped in both studies. In regard to *IL13*, four SNPs were in common between the two studies, namely rs20541, rs1800925, rs1295685, and rs1881457. In both the IOW and PAPA studies the four SNPs are in medium to high linkage disequilibrium (D′ values: IOW: 52 – 99; PAPA: 53 – 99; r^2^ values: IOW: 26 – 97; PAPA: 26 – 99). This observation of elevated linkage disequilibrium between SNPs in *IL13* gene had been reported previously [17,27]. Due to the moderate to high linkage disequilibrium between the four SNPs we restricted our analysis to the functional (non-synonymous) SNP rs20541, which in turn is also a proxy of other SNPs in the region. Deviation from Hardy-Weinberg Equilibrium (HWE) was tested by conducting χ^2^ goodness-of-fit test for genotype frequencies in both study populations. A p-value < 0.05 was used as an indicator for possible violation of HWE.

Odds ratios are likely to overestimate the risk ratios due to the fact that the prevalence of eczema is common (> 10%) in most of the strata used in our analysis [28]. To evaluate associations between SNP genotypes and eczema, log-binomial regression was applied to estimate risk ratios (RRs) and 95% confidence intervals (CIs). Exponentiating coefficients associated with explanatory variables (i.e., SNP genotypes) estimated by log-binomial regression with a log link function allowed us to directly estimate RRs [28]. To estimate the overall effect of SNP genotypes on eczema, which was repeatedly measured at 1-or-2, 4, 10, and 18 years in the IOW study, we applied generalized estimating equation (GEE) method [29]. The GEE approach accounts for the correlated observations and the within-child effect by employing a covariance matrix solved through an iterative estimating process based on a working correlation matrix [29]. Log-binomial models were estimated using the GENMOD procedure in SAS 9.2 (SAS, Gary, NC, USA). In all statistical models age and sex were included as potential covariates and a p-value < 0.05 was used to indicate statistical significance.

In a first exploratory step, the associations between genotypes of the two SNPs and eczema were explored using co-dominant, dominant, recessive, and additive genetic models. We applied log-binomial regression models to obtain RRs for eczema relative to SNPs in *IL13* and *STAT6* separately. Although, results of this step are not the focus of this article, controlling for false-positive findings was performed using the false discovery rate (FDR) method [30]. However, the aim of this study is to test the statistical interaction of the two SNPs for the risk of eczema. Therefore, in the discovery study using the IOW cohort, log-binomial regression models {RR = exp(β_1_ × *IL13* + β_2_ × *STAT6* + β_3_ × [*IL13* × *STAT6*])} were conducted to evaluate the statistical interaction between the SNPs of the two genes under four genetic models (co-dominant, dominant, recessive, and additive). To control for false-positive interactions, the FDR method was applied to obtain adjusted p-values associated with the interaction terms. In the replication study, i.e. the PAPA study, we only tested the statistical interaction between the two SNPs under the genetic model that gained statistical significance in the discovery study. Following the concept of replication, no correction for multiple testing was needed in the replication study (one model was tested for interaction). The interaction term (β_3_ × [*IL13* × *STAT6*]) was used to estimate the additional effect of two co-occurring risk factors on the health outcome above and beyond their individual effects. The term “combined effect” was used to describe the joint impact of two individual risk factors plus their interaction on the occurrence of the outcome (eczema). We also applied an alternate approach to estimate the combined effect of both risk factors; we compared the risk of eczema in individuals who have both risk factors (*IL13* and *STAT6* risk genotypes) to the risk of individuals who have neither (*IL13* and *STAT6* wild genotypes). This approach was referred to as the “combined effect – stratification”.

## Results

The period prevalence of eczema in the IOW study ranged from 11.9% to 14.2% during the first 18 years of life. In the PAPA study, 10.3% of the young adults (median age of 20 years) were affected by eczema (Table 1). In both studies, genotype frequencies of *IL13* (rs20541) and *STAT6* (rs1059513) polymorphisms were concordant with Hardy-Weinberg equilibrium. Moreover, allele frequencies were comparable between the two studies and with data from the SNP database (dbSNP) for populations of European ancestry.

**Table 1:** Characteristics of the IOW and PAPA study populations and description of the SNPs investigated

**Attribute**	**IOW study % (n/total)**	**PAPA study % (n/total)**
**Age range in years**	1 to 18	18 to 30
**Gender**		
Male	51.2 (786/1536)	48.3 (698/1445)
Female	48.8 (750/1536)	51.7 (747/1445)
**Eczema prevalence**		10.3 (147/1435)
1-or-2 yr	14.2 (196/1377)	
4 yr	11.9 (145/1214)	
10 yr	13.7 (186/1359)	
18 yr	12.3 (161/1307)	
***IL13 *(rs20541)**		
GG	65.0 (747/1149)	68.7 (973/1416)
AG	31.7 (364/1149)	28.3 (400/1416)
AA	3.3 (38/1149)	3.0 (43/1416)
MAF (allele A)	0.192	0.172
HWE p-value	0.450	0.861
***STAT6 *(rs1059513)**		
TT	79.6 (921/1157)	76.9 (1090/1418)
TC	19.5 (225/1157)	21.8 (309/1418)
CC	0.95 (11/1157)	1.3 (19/1418)
MAF (allele C)	0.108	0.122
HWE p-value	0.501	0.693

MAF = Minor allele frequency.

HWE = Hardy-Weinberg equilibrium.

In the first exploratory step analyzing main effects only, under a co-dominant model, the AG and AA genotypes of *IL13* rs20541 were not statistically significantly associated with eczema when compared to the reference genotype GG in both populations (Table 2). In the IOW study, the homozygous genotype for the minor allele CC of *STAT6* rs1059513 was significantly associated with eczema in the repeated measurements analysis when compared to the non-risk genotype TT (RR = 2.21; 95% CI: 1.05 – 4.66). However, this association did not remain statistically significant after penalizing its p-value for multiple testing (adjusted p-value = 0.857). Similarly, under the co-dominant model *STAT6* rs1059513 was not associated with eczema in the PAPA study. In the dominant model, individuals carrying the AG or AA genotype of *IL13* SNP rs20541 did not have increased risk of eczema when compared to those with the GG genotype in either population (Table 2). Similarly, there was no main effect of *STAT6* SNP rs1059513 and eczema in the dominant model in either population.

**Table 2:** **Associations of *IL13 *and *STAT6 *SNPs genotypes with eczema: results from IOW and PAPA studies**

**Gene (SNP rs no.)**	**Model**	**Genotype**	**Eczema % (n/total)**	**RR**^**†**^	**95% CI**
***IL13 *(rs20541)**					
IOW study^‡^	Co-dominant	GG	12.8 (357/2781)	1.00	–
		AG	14.3 (194/1357)	1.10	0.88 – 1.38
		AA	16.9 (24/142)	1.37	0.83 – 2.25
			p-trend^*^	*0.08*	
	Dominant	GG	12.8 (357/2781)	1.00	–
		AG/AA	14.5 (218/1499)	1.13	0.91 – 1.40
PAPA study	Co-dominant	GG	9.5 (92/967)	1.00	–
		AG	12.3 (49/397)	1.27	0.92 – 1.76
		AA	11.6 (5/43)	1.14	0.49 – 2.68
			p-trend^*^	*0.147*	
	Dominant	GG	9.5 (92/967)	1.00	–
		AG/AA	12.3 (54/440)	1.26	0.91 – 1.73
***STAT6 *(rs1059513)**					
IOW study^‡^	Co-dominant	TT	13.6 (467/3438)	1.00	–
		TC	12.8 (106/830)	0.92	0.70– 1.20
		CC	28.6 (12/42)	2.21	1.05 – 4.66^$^
			p-trend^*^	*0.54*	
	Dominant	TT	13.6 (467/3438)	1.00	–
		TC/CC	13.5 (118/872)	0.98	0.75 – 1.27
PAPA study	Co-dominant	TT	9.8 (106/1082)	1.00	–
		TC	12.3 (38/308)	1.33	0.94 – 1.88
		CC	10.5 (2/19)	1.16	0.31 – 4.30
			p-trend^*^	*0.243*	
	Dominant	TT	9.8 (106/1082)	1.00	–
		TC/CC	12.2 (40/327)	1.32	0.93 – 1.86

^†^ Association adjusted for gender and age at follow up.

^*^ Based on Cochran-Armitage Trend Test.

^‡^ Association results for the IOW study were based on repeated measurement analysis (GEE models), which utilized data collected at the 1-or-2, 4, 10, and 18 years follow-ups.

^$^ The association was not statistically significant after adjusting for multiple testing (adjusted p-value = 0.857).

In the discovery step, to determine whether *IL13* (rs20541) and *STAT6* (rs1059513) SNP genotypes have an interactive effect on the risk for eczema, four genetic models (co-dominant, dominant, recessive, and additive) were used to test for statistical interaction (*IL13* × *STAT6*) in the IOW study. After adjusting for multiple testing, using FDR method, only the interaction term under the dominant genetic model remained statistically significant (adjusted *P*_interaction_ = 0.046; Table 3). In the replication step using the PAPA study, we only tested the interaction term between (*IL13* × *STAT6*) under the dominant genetic model. In line with the IOW study results, this interaction was replicated in the PAPA study (*P*_interaction_ = 0.037; Table 3). In addition to the interaction term, the combined effect, defined as the risk of eczema among individuals with AG/AA (rs20541) and TC/CC (rs1059513) genotypes compared to the risk of eczema among individuals with GG (rs20541) and TT (rs1059513) genotypes, was estimated using two approaches. The modeling approach took into account the main effects of each SNP plus their interaction effect using the whole sample. A stratification approach was applied using stratification method in which individuals with risk genotypes for both SNPs were grouped together and those with non-risk genotypes formed the referent group. In the IOW study, the combined effect based on the modeling approach yielded a 1.41-fold trend for increased risk of eczema which was borderline for being statistically significant (*P* = 0.063). The stratification method showed that the combined effect associated with having risk genotypes in both SNPs demonstrated statistical significance (RR = 1.52; *P* = 0.028). Concordant with results from the IOW study, in the PAPA study, both the modeling and stratification approaches estimated a significant combined effect of an approximately 2-fold increased risk of having eczema (Modeling: *P* = 0.003; Stratification: *P* = 0.002; Table 3).

**Table 3:** **Interaction and combined effects of *IL13 *(rs20541) and *STAT6 *(rs1059513) single nucleotide polymorphisms on eczema risk: results from IOW and PAPA studies**

**IOW (discovery) study**^**† **^**– Dominant model**				
**Variable**	**Coefficient**	**RR**^**$**^	**95% CI**	**p-value**
*IL13* (AG/AA vs. GG)	−0.008	0.99	0.53 – 1.09	0.952
*STAT6* (TC/CC vs. TT)	−0.278	0.76	0.77 – 1.27	0.135
Interaction (*IL13* × *STAT6*)	0.630	1.88	1.10 – 3.19	0.046^£^
Combined effect – Modeling^*^	0.344	1.41	0.98 – 2.03	0.063
Combined effect – Stratification^¶^	0.418	1.52	1.05 – 2.20	0.028
**PAPA (replication) Study – Dominant model**				
*IL13* (AG/AA vs. GG)	−0.043	0.96	0.64 – 1.43	0.833
*STAT6* (TC/CC vs. TT)	−0.025	0.98	0.60 – 1.58	0.920
Interaction (*IL13* × *STAT6*)	0.754	2.13	1.05 – 4.31	0.037
Combined effect – Modeling^‡^	0.686	1.99	1.27 – 3.11	0.003
Combined effect – Stratification^¶^	0.698	2.01	1.29 – 3.12	0.002

^†^ Association results for the IOW study were based on repeated measurement analysis (GEE models), which utilized data collected at the 1-or-2, 4, 10, and 18 years follow-ups.

^$^ Association adjusted for gender and age at follow up.

^£^ Adjusted for multiple testing using the false discovery rate method p-value.

^*^ Combined effect – Modeling RR was estimated as: RR = exp(−0.008 – 0.278 + 0.630).

^‡^ Combined effect – Modeling RR was estimated as: RR = exp(−0.043 – 0.025 + 0.754).

^¶^ Combined effect – Stratification RR was estimated by pooling together individuals with risk genotypes for both SNPs and those with non-risk genotypes formed the referent group.

## Discussion

Eczema is a multifactorial disorder that is believed to be the result of a complex interaction between genetic, environmental, and immunological factors. The high heritability and clustering of eczema within families highlight the importance of the genetic component in disease pathogenesis [31]. Genes that regulate the epidermal barrier formation and immunological response have been implicated in eczema development [9,31,32]. This study adds to the current knowledge of genetic susceptibility by demonstrating for the first time an interactive effect between SNPs in *IL13* (rs20541) and *STAT6* (rs1059513) on the occurrence of eczema in two independent samples. Our results emphasize the importance of accounting for the effect of multiple genes (epistasis/gene-gene interaction) when assessing the genetic contribution in the development of complex diseases, such as eczema.

The current study investigated the association of SNPs in *IL13* (rs20541) and *STAT6* (rs1059513) genes separately and jointly (gene-gene interaction) on the risk for eczema in two independent population-based studies. The SNP rs20541 was not associated with eczema in either the IOW nor PAPA studies. Similar results were obtained for the SNP rs1059513, which showed no association in both the IOW and PAPA studies. However, a consistent significant statistical interaction under a dominant genetic model between *IL13* (rs20541) and *STAT6* (rs1059513) on the risk of eczema was demonstrated in both studies. Participants in the IOW study who carried the risk genotypes for both SNPs had a 1.52-fold increased risk for eczema during the first 18 years of life. Similarly, in the PAPA population, those with the risk genotypes for both SNPs had a 2.01-fold higher risk of eczema than those with wild-type genotypes for both SNPs. Our investigation demonstrates an important combined effect of genetic variants within *IL13* and *STAT6* genes on eczema risk.

The longitudinal design of the Isle of Wight birth cohort study that covered the first 18 years of life is a major strength of our study. Throughout all assessments, the proportion of participation remained high, which ruled-out a major bias due to loss-to-follow up. Misclassification of eczema cases is minimal since a high proportion of subjects showed typical manifestation of eczema in usual locations (antecubital or popliteal fossae, ankles, face or neck for 97% at 1 year, 91% at 2 years, 75% at 4 years, 86% at 10 years and 76% at 18 years) [33]. In addition, the prevalence of eczema in our cohort (13.7% at age 10 years and 12.3% at age 18 years) is comparable to results from the International Study of Asthma and Allergies in Childhood aged 13–14 years in the United Kingdom (UK) (14.7% phase one and 10.6% phase three) and other studies conducted in the UK [34-36].

A major strength of the PAPA study was the large number of young adult volunteers within a relatively narrow age range. Participants were comprehensively phenotyped with relation to clinical allergic/atopic phenotypes. Eczema was self-reported by validated questionnaire which is considered as robust for epidemiological investigations [26]. Self-reporting might have led to some misclassification but this was minimized by the same physician being present when completing questionnaires (GAD) to answer question on eczema. The eczema prevalence (10.3%) in young adults (median age 20 years) in the study is comparable to European prevalence in a multicentre study (8.8%) in a slightly older age group (27–35 years), indicating that any potential misclassification was minor and that our unselected population was a representative sample [37]. In both studies, participants were not aware of their genetic status, nor were the medical investigators. Hence misclassification was non-differential, which is no threat to validity and tends to reduce the relative estimates of the relative risks.

The minor allele frequency (MAF) of rs20541 was comparable across the two study populations (IOW: 19.2%; PAPA: 17.2%). Moreover, allele frequencies were comparable with data from the SNP database (dbSNP) for populations of European ancestry, which ranged from 14% to 23% based on the HapMap project data. In regard to rs1059513, the MAF was similar between the two study populations (IOW: 10.8%; PAPA: 12.2%) and within the range for populations of European ancestry (6% to 18%).

Genetic variants in the *IL13* gene region have shown association with asthma, eczema, allergic rhinitis, and increased IgE levels in multiple studies of diverse race/ethnic backgrounds [16,27]. Similarly, SNPs in the genomic region of the *STAT6* gene had been reported to be associated with allergic phenotypes, especially regulation of IgE production [22,38]. However, the previously noted associations were not consistent throughout the literature. Such inconsistencies that hamper genetic association studies can be explained, at least in part, by population diversity, phenotype heterogeneity, lack of independent replication of genotype-phenotype associations, or not accounting for possible gene-gene or gene-environment interactions. Furthermore, the small to modest effect sizes (e.g., risk/odds ratios from 1.1 to 1.5) associated with genetic variants on the risk of complex diseases found in GWAS can possibly be improved if interaction of multiple variants is considered [39,40]. In this report we have investigated the interactive effect between rs20541 and rs1059513 on eczema risk for the following reasons: (i) the functional variant (rs20541) of *IL13* was associated with increased IL-13 biological activity [27], (ii) *IL13* rs20541 leads to increased activation of *STAT6*[17], (iii) *STAT6* rs1059513 is associated with IgE dysregulation [22], (iv) the interaction between SNPs in *IL13* and *STAT6* on the risk for eczema have not, to our knowledge, been studied previously, however, (v) studies have shown that genetic variants within *IL13* and *STAT6* have interactive effects on IgE synthesis [41] and asthma risk [42]. Genotyping of these two SNPs in both the IOW and PAPA studies facilitated the replication of our findings.

A limitation to our study is that only two SNPs (one in each gene) were investigated. However, as previously mentioned, their importance in the pathogenesis of allergic phenotypes is well established [10,17,22,27]. Therefore, results of this report should stimulate future studies to incorporate a wider range of SNPs as such an approach might reveal larger effect sizes. While no formal tests of population stratification were carried out as data from ancestry informative makers were not available, both study populations were of UK Caucasian ancestry, which reduces the chance that the observed associations are a result of population stratification. Although the two study populations differed in their age group structure (IOW study: longitudinal assessment covering the first 18 years of life; PAPA study: participants aged 18 to 30 years), we were able to find comparable interaction results across the two studies. Slightly different epidemiologic methods, 12-month period prevalence (IOW) and point prevalence (PAPA), were used to measure disease occurrence. However, the comparable proportion of individuals with eczema in the IOW study (12.3% at 18 years) and in the PAPA study (10.3% between 18 to 30 years) is a sound indication that phenotypic heterogeneity is not an issue.

The use of log binomial regression to estimate risk ratios could raise issues since it is less stable than logistic regression [43]. However, we have compared the performance of both methods (data not shown) and decided to present estimates obtained from the log binomial regression due to the similar results provided by both methods. The advantage of using log binomial regression is that it allows direct estimation of adjusted risk ratios; whereas, logistic regression estimates odds ratios which is vulnerable to overestimate the relative risk when the prevalence of the outcome is not rare in all analyzed strata of the outcome [28].

Eczema is a complex disease in which both immunological imbalances and dysfunctional skin barrier play a major role in its pathogenesis [44]. The discovery of loss-of-function variants in the filaggrin gene and their successful association with eczema in multiple studies has shed light on the importance of skin barrier genetics. However, inflammatory Th2 cytokines, such as IL-13 and IL-4, have been shown to modulate the expression of keratinocyte genes leading to epidermal thickening and impaired skin barrier [45]. Furthermore, it has been shown that the down-regulation of epidermis-differentiation related genes by Th2 cytokines is a STAT6-dependent mechanism [6]. Therefore, Th2 cytokines could contribute to the immunologic disturbance and dysfunctional skin barrier that characterize eczema.

## Conclusions

The current study demonstrates that gene-gene interaction (epistasis) between *IL13* and *STAT6* polymorphisms contributes significantly to the genetic determination of eczema in two independent British epidemiological studies. This work shows that analysis of epistatic effects, in addition to individual SNP analysis, will provide a more comprehensive picture of how genetic variants of genes sharing a biological pathway may interact to produce complex disorders, such as eczema. Therefore, future studies should move beyond the one SNP by one SNP approach taken so far, and simultaneously interrogate multiple genes within a biological pathway to shed more light on the contribution of genetic susceptibility.

## Abbreviations

IOW: Isle of Wight; PAPA: Poblogaeth Asthma Prifysgol Abertawe; STAT6: Signal transducer and activator of transcription 6; IL4: Interleukin 4; IL13: Interleukin 13; SNP: Single nucleotide polymorphism; RR: Risk ratio; CI: Confidence interval; FDR: False discovery rate.

## Competing interests

The authors declare that they have no competing interests.

## Authors’ contributions

AHZ performed the statistical analyses, participated in the design of the epidemiological analyses, and drafted the manuscript. SE, JMH, and GAD performed genetic analysis and revised the manuscript. EMS participated in genetic analysis and carried out molecular genetics experiments. WK participated in statistical analysis and drafting the manuscript. HZ participated in statistical analysis. SHA and GAD carried out the clinical assessments and revised the manuscript. WK, GAD, SHA, JMH, SE, and JWH contributed to the study concept and design and acquisition of data. All authors read and approved the final manuscript.

## Pre-publication history

The pre-publication history for this paper can be accessed here:

http://www.biomedcentral.com/1471-2350/14/67/prepub

## Supplementary Material

Additional file 1: Table S1 Quality control indices for genotyping *IL13* rs20541 and *STAT6* rs1059513 in the Isle of Wight population and PAPA study population. Click here for file
