# Supplementary material for: Interactive effect of STAT6 and IL13 gene polymorphisms on eczema status: results from a longitudinal and a cross-sectional study
Source: BMC Med Genet. 2013 Jul 2;14:67. doi: 10.1186/1471-2350-14-67 (PMC3700873; doi:10.1186/1471-2350-14-67)
Supplement: Additional file 1: Table S1 — Quality control indices for genotyping IL13 rs20541 and STAT6 rs1059513 in the Isle of Wight population and PAPA study population. [file 1471-2350-14-67-S1.pdf]

Table S1. Quality control indices for genotyping *IL13* rs20541 and *STAT6* rs1059513 in the Isle of Wight study population and PAPA study population

**Quality Control Index**	***IL13* rs20541**		***STAT6* rs1059513**	
	IOW	PAPA	IOW	PAPA
SNP call rate	0.966	0.989	0.975	0.990
A/A frequency	0.649	0.678	0.796	0.777
A/B frequency	0.315	0.288	0.195	0.210
B/B frequency	0.036	0.034	0.009	0.013
Minor allele frequency	0.193	0.178	0.106	0.118
HWE χ^2	0.143	0.812	0.702	0.895
HWE χ^2 p-values[†]	0.704	0.368	0.402	0.344
[1]50% GenCall score	0.771	NA	0.823	NA
[2]10% GenCall score	0.770	NA	0.823	NA
[3]Cluster sep	0.592	NA	0.623	NA

[†] A p-value < 0.05 is an indication of possible violation of HWE

[1]50% GenCall score represents the 50% percentile of the distribution of GenCall scores for SNPs across all samples

[2]10% GenCall score represents the 10% percentile of the distribution of GenCall scores for SNPs across all samples

[3]Cluster sep measures the separation between the three genotype clusters in the theta dimension and varies from 0.0 to 1.0.
